# A comparison of the effects of agricultural pesticide uses on peripheral nerve conduction in China

**DOI:** 10.1038/s41598-018-27713-6

**Published:** 2018-06-25

**Authors:** Chao Zhang, Yiduo Sun, Ruifa Hu, Jikun Huang, Xusheng Huang, Yifan Li, Yanhong Yin, Zhaohui Chen

**Affiliations:** 1 School of Humanities and Social Sciences, Beijing Institute of Technology, 5 South Zhongguancun Street, Beijing, 100081 China; 2 School of Management and Economics, Beijing Institute of Technology, 5 South Zhongguancun Street, Beijing, 100081 China; 3 School of Advanced Agricultural Sciences and China Center for Agricultural Policy, Peking University, 5 Yiheyuan Road, Beijing, 100871 China; 4 Department of Neurology, Chinese PLA General Hospital, 28 Fuxing Road, Beijing, 100853 China; 5 Department of Neurology, the First Affiliated Hospital of PLA General Hospital, No. 51 Fucheng Road, Beijing, 100048 China

## Abstract

Evidence on the adverse effects of agricultural pesticide use by farmers under the actual field conditions on their peripheral nerve conduction in China is limited. This study was to investigate the association of agricultural pesticide use with the abnormalities of farmers’ peripheral nerve conduction based on two rounds of conventional nerve conduction studies. The level of pesticide exposure was assessed by measuring total amount of pesticides used by farmers in 2012. The logistic and negative binomial regression analyses were performed on a cohort study of 218 farmers. Results show that agricultural use of neither glyphosate nor non-glyphosate herbicides was not found to induce the abnormalities of farmers’ peripheral nerve conduction. However, agricultural use of organophosphorus compounds was significantly associated with increased risk of demylination disease of peripheral nerve conduction described by the reduced velocity. Moreover, the use of organonitrogen compounds by farmers would not only increase risk of demylination disease but axonal damages described by the reduced amplitude. By contrast, agricultural uses of organosulfur and pyrethroid compounds would not induce the abnormalities of farmers’ peripheral nerve conduction. The findings demonstrated the importance of developing health-friendly pesticides to replace organophosphorus and organonitrogen insecticides and fungicides in China.

## Introduction

Many studies agree that agricultural pesticide use contributes to the reduction of crop loss due to pest infestation^1,2^. However, it also generates some major negative effects, including the spread of resistance, environmental pollution and health problems^3,4^. The growing epidemiological literature has paid much attention to the adverse human health effects of pesticide exposure^3,4^. In sum, pesticide exposure has been repeatedly associated with the pathological changes of reproductive organs, abnormal fetal development and reproductive dysfunction^5^. In addition, pesticide exposure would also induce the impairment effect on human’s digestive, respiratory, endocrine, urinary and cardiovascular systems^4^. Moreover, pesticide exposure has even been reported as carcinogenicity^3^.

Since most widely used pesticides are nerve agents^6^, a large number of epidemiological studies argued that pesticide exposure would induce peripheral nerve conduction dysfunction, cognitive disorder, intelligence impairment and Parkinson’s diseases^3,4,7^. However, most studies merely made a comparison of the neurological outcomes between the exposed and unexposed groups^3^. In addition, little is known about whether and how multiple types of pesticides used by farmers under actual field conditions induces adverse effects on their peripheral nerve conduction. Since most farmers in China apply multiple pesticides (including herbicides, insecticides and fungicides), it is crucial to identify the specific association of agricultural use of each type of pesticides with peripheral nerve conduction function. A previously published study estimated the marginal changes in parameters of farmers’ peripheral nerve conduction induced by one-year exposure to different types of pesticides^8^. However, there is still no clear-cut evidence that how each type of pesticides used by farmers under the actual field conditions associates with the abnormalities of peripheral nerve conduction.

To address this gap, this study aims to investigate the association of each type of pesticides used by farmers with the abnormalities of peripheral nerve conduction in China. Both multiple logistic and negative binomial regression analyses are utilized. Results show that neither glyphosate nor non-glyphosate herbicides was associated with the abnormalities of farmers’ peripheral nerve conduction. However, agricultural uses of organophosphorus and organonitrogen insecticides and fungicides were found to increase risk of demylination disease described by the reduced nerve conduction velocity and axonal damages described by the reduced amplitude.

## Materials and Methods

### Study sample

The sampling procedure in the cohort study has been previously described^6,8^. In sum, we selected Guangdong, Jiangxi and Hebei as the study areas due to their different levels of agriculture pesticide use intensity. In 2011, pesticide use intensity in Guangdong, Jiangxi and Hebei was 25.0, 18.2 and 9.5 kilograms per hectare (kg/ha), respectively^9^.

All the sampled farmers were selected according to the principle of random sampling. In brief, two counties were chosen within each province, and two villages within each county were then selected. In each sampled village, the sampling frame was constructed from the full farm household list provided by the village leaders. In each village, 20–25 households were chosen. In principle, the family member mainly engaged in pesticide use was enrolled to construct the sample. As a result, 246 farmers were initially chosen. All the sampled farmers were informed of the study objective. However, 28 out of them were excluded since they were absent from the conventional nerve conduction studies, or failed to provide detailed information of pesticide use. As a result, 218 farmers finally remained.

### Conventional nerve conduction studies

In order to obtain the parameters of peripheral nerve conduction, two rounds of conventional nerve conduction studies were conducted for the sampled farmers in 2012. The first round of conventional nerve conduction studies was conducted at the beginning of the planting season in March in three provinces, while the second round was conducted prior but close to the end of crop harvest in August in Jiangxi and Hebei but in December in Guangdong. Note that the parameters from the second round of conventional nerve conduction studies were of interest, while those of the first round were used to describe the baseline status.

In this study, 22 parameters of peripheral nerve conduction were examined using the surface electrodes with standard placement. It should be noted that the majority of farmers in China undertake an immense amount of manual work that might induce peripheral nerve injury in their fields, and such peripheral nerve injury induced by manual work is difficult to control. In the context, the examination was performed on the non-dominant side of the sampled farmers to avoid the potential interference in the identification of the effects of pesticide use on peripheral nerve conduction. Since the accuracy of the conventional nerve conduction studies might be affected by limb temperature, both upper and lower limbs of the sampled farmers were warmed so that the temperatures of upper and lower limbs could maintain in the range of 32–34 °C and 30–33 °C, respectively.

In the study, two upper limb nerves (namely median and ulnar nerves) and three lower limb nerves (namely tibial, common peroneal and sural nerves) were examined. In detail, the motor conduction velocity, distal motor latency and amplitude of the proximal and distal compound muscle action potential of the median, ulnar, tibial and common peroneal nerves, as well as the sensory conduction velocity and amplitude of the sensory nerve action potential of the median, ulnar and sural nerves, were examined in both rounds of conventional nerve conduction studies. It should be noted that the metric unit of the nerve conduction velocity, distal motor latency and amplitude is meter per second (m/s), millisecond (ms) and millivolt (mV), respectively.

To obtain the dependent variables, we generated seven dummy variables for the abnormalities of the nerve conduction velocity, motor conduction velocity, sensory conduction velocity, distal motor latency, amplitude of the action potential, amplitude of the compound muscle action potential and amplitude of the sensory nerve action potential (see Table S1 for the normal range of each parameter). In the case of the motor conduction velocity, for example, we defined a dummy variable equaling one if one and more parameters of the motor conduction velocity fell out of the normal range, and zero otherwise. Afterwards, we also generated seven count variables for the abnormalities of the nerve conduction velocity, motor conduction velocity, sensory conduction velocity, distal motor latency, amplitude of the action potential, amplitude of the compound muscle action potential and amplitude of the sensory nerve action potential by aggregating the number of the abnormal parameters with each group.

The Ethics Committee of Chinese PLA General Hospital approved the study, and Beijing Institute of Technology organized the sample selection. The methods in the study were carried out in accordance with the ethical standards and guidelines of the Ethics Committee of Chinese PLA General Hospital. All the sampled farmers provided *ex ante* informed and written consents.

### Estimation of agricultural pesticide use

Agricultural pesticide use was measured by the actual amount of pesticides used by the sampled farmers between two rounds of conventional nerve conduction studies in 2012. Hence, the duration for measuring agricultural pesticide use by the sampled farmers was confined to 2012 (one year). All the sampled farmers were requested to record each pesticide use in detail. Briefly, once a sampled farmer sprayed pesticides, they should record the chemical name, active ingredient percentage and amount of each pesticide product, as well as the date and duration of each spray. In order to guarantee the correctness and completeness of records, a number of training sessions on making proper records were organized during the study period in 2012. In addition, the enumerators were arranged to check the sampled farmers’ records every other week, and all containers of pesticides were kept for the semimonthly check.

In this study, all pesticides used by the sampled farmers were firstly divided into two parts: (1) herbicides, and (2) insecticides and fungicides. Then, herbicides were categorized as glyphosate, and non-glyphosate herbicides. Insecticides and fungicides were divided into five classes, including organophosphorus, organonitrogen, organosulfur, pyrethroid and other compounds. Thus, we were able to investigate the association of agricultural uses of those different classes of pesticides with the abnormalities of farmers’ peripheral nerve conduction. The level of agricultural use of each class of pesticides was the product of the amount of pesticide product and the corresponding active ingredient percentage. In this study, the basic metric of agricultural pesticide use is kilogram (kg).

### Selection of confounding factors

In this study, a number of confounding factors were also included o obtain the net association between agricultural pesticide use and the abnormalities of peripheral nerve conduction. A questionnaire was designed to obtain information about farmers’ demographic and lifestyle characteristics, agricultural production, and history of pesticide use and poisoning. Specifically, age, gender, smoking habit, alcohol consumption, whether adopting protective measures (including wearing masks, gloves, and clothes with long sleeves), and whether suffering from diabetes mellitus (a major factor causing polyneuropathy) were selected as the confounding factors. Body mass index (BMI) was also calculated for each sampled farmer using the height and weight.

### Statistical analyses

Descriptive analyses were firstly used to summarize farmers’ demographic and lifestyle characteristics as well as agricultural pesticide use. Continuous variables (such as age and BMI) were described by the mean with standard deviation, while dummy variables (such as gender, smoking habit, alcohol consumption, adoption of protective measures, and diabetes mellitus) were described by the number with percentage.

Since most farmers using herbicides also use insecticides and fungicides, a crucial technical issue is to sort out the effect of agricultural use of each class of pesticides on the abnormalities of peripheral nerve conduction. Multiple regression analysis could control for the effects of not only the confounding factors but also different classes of pesticides, which empowered us to sort out the specific effect of agricultural use of each class of pesticides on the abnormalities of peripheral nerve conduction. The model was constructed as:

1 $${\boldsymbol{NP}}=f({\boldsymbol{Pesticide}},{\boldsymbol{Characteristics}},{\boldsymbol{BaselineNP}},{\boldsymbol{Regions}})$$

where the dependent variable ***NP*** denotes the dummy or count variables for the abnormalities of peripheral nerve conduction from the second round of conventional nerve conduction studies. In terms of the independent variables, ***Pesticide*** is a vector of agricultural uses of seven classes of pesticides, including herbicides (glyphosate and non-glyphosate herbicides), and insecticides and fungicides (organophosphorus, organonitrogen, organosulfur, pyrethroid and other compounds). ***Characteristics*** is a vector of farmers’ demographic and lifestyle characteristics, such as age, gender, BMI, smoking habit, alcohol consumption, whether adopting protective measures and whether suffering from diabetes mellitus. ***BaselineNP***, a variable corresponding to ***NP***, describes the baseline status of peripheral nerve conduction. In addition, a vector of province dummies, ***Regions***, are also included to control for regional effect.

Logistic and negative binomial regression analyses were used to estimate the odds ratios (ORs) and incidence rate ratios (IRRs) for the dummy and count dependent variables, respectively. Note that a 95% confidence interval (CI) was estimated for each independent variable.

All statistical tests were two-sided, and *p*-value < 0.05 was considered as statistically significant. It should be noted that values of ORs or IRRs > 1.0 suggests positive associations between agricultural pesticide use and the abnormalities of peripheral nerve conduction. Stata version 13.1 was used (StataCorp LP, College Station, Texas) for all statistical analyses.

## Results

### Demographic and lifestyle characteristics

Table 1 presents farmers’ demographic and lifestyle characteristics. Of 218 sampled farmers, the mean age was 51.6 years with a nearly ten-year standard deviation. 90 of these farmers were under 50 years old, while 17 of them were above 65 years old. The number of male farmers was 161, accounting for about three-fourths. The mean value of BMI for the sampled farmers was about 23.4 kg/m^2^. Almost half of the sampled farmers consumed cigarette (47.7%) and alcohol (43.1%) in their daily life. Only 27 out of the sampled farmers reported that they adopted some crude protective measures, such as wearing masks, gloves and clothes with long sleeves, when using pesticides in crop field. There were 14 (6.4%) out of the sampled farmers suffering from diabetes mellitus.

**Table 1:** Description of farmers’ demographic and lifestyle characteristics.

Characteristics	Mean	Standard deviation	Number of farmers	Percentage (%)
Age (year)	51.6	10.1		
<50	41.7	5.9	90	41.3
50–65	57.1	4.1	111	50.9
>65	68.6	1.7	17	7.8
Gender				
Male			161	73.9
Female			57	26.1
Body mass index (BMI) (kg m^−2^)	23.4	3.4		
<18	17.5	0.3	8	3.7
18–25	21.9	1.7	146	67.0
>25	27.7	2.2	64	29.4
Smoking habit				
Yes			104	47.7
No			114	52.3
Alcohol consumption				
Yes			94	43.1
No			124	56.9
Adoption of protective measures				
Yes			27	12.4
No			191	87.6
Diabetes mellitus				
Yes			14	6.4
No			204	93.6

The number of the sampled farmers is 218.

### Summary of agricultural pesticide use

**Figure 1:**
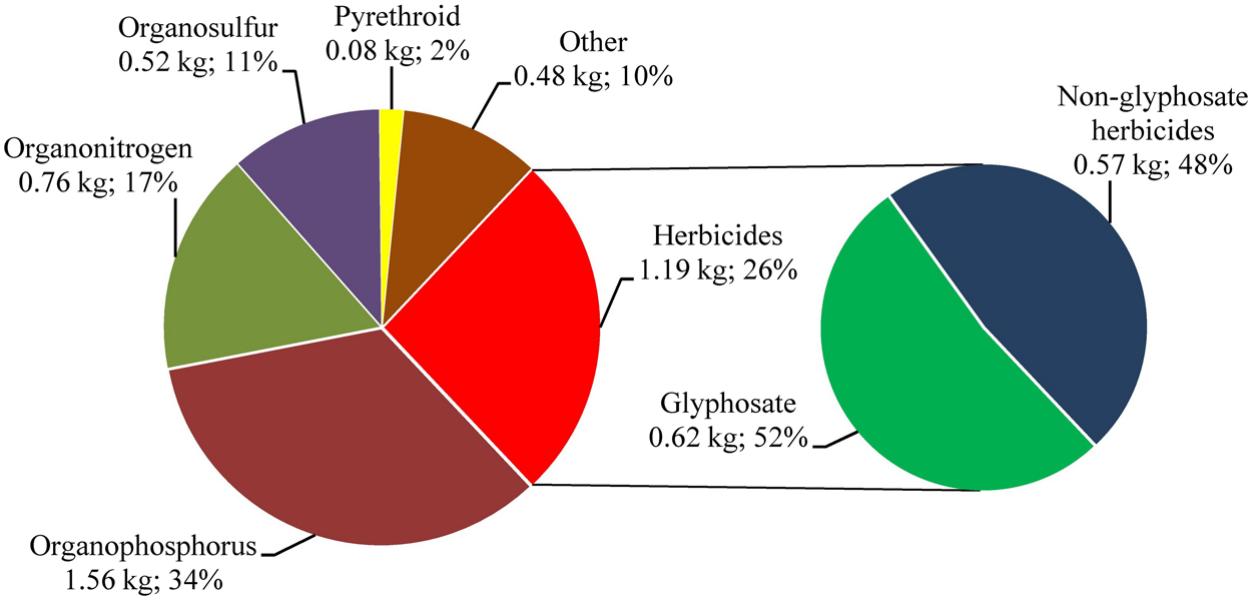
Summary of agricultural use of each class of pesticides by the sampled farmers. Figures before and after the semicolon are the average amount and percentage of each class of pesticides in the total used pesticides, respectively. The number of the sampled farmers is 218.

Agricultural pesticide use of the sampled farmers is reported in Fig. 1. In sum, about 4.58 kg of pesticides were used per farmer in 2012. Among these pesticides were 1.19 kg of herbicides and the other 3.39 kg of insecticides and fungicides.

On average, the amount of glyphosate used by each farmer was about 0.62 kg that accounted for more than half of the total herbicide use. By contrast, the amount of non-glyphosate herbicides used was 0.57 kg per farmer. Note that roughly 0.44 kg (or 77%) of non-glyphosate herbicides used by the sampled farmers were organonitrogen compounds, such as paraquat, atrazine and acetochlor.

In terms of insecticides and fungicides, as shown in Fig. 1, the amount of organophosphorus compounds used by each farmer was about 1.56 kg. It was obvious that organophosphorus compounds accounted for the largest proportion of pesticides used by the sampled farmers. By contrast, agricultural uses of other classes of insecticides and fungicides were much less. The amount of organonitrogen compounds, for example, was roughly 0.76 kg per farmer. Moreover, only 0.52 kg of organosulfur compounds were used by each farmer on average. Agricultural use of pyrethroid insecticides was the least, even less than 0.1 kg per farmer.

### Summary of the abnormalities of peripheral nerve conduction

**Figure 2:**
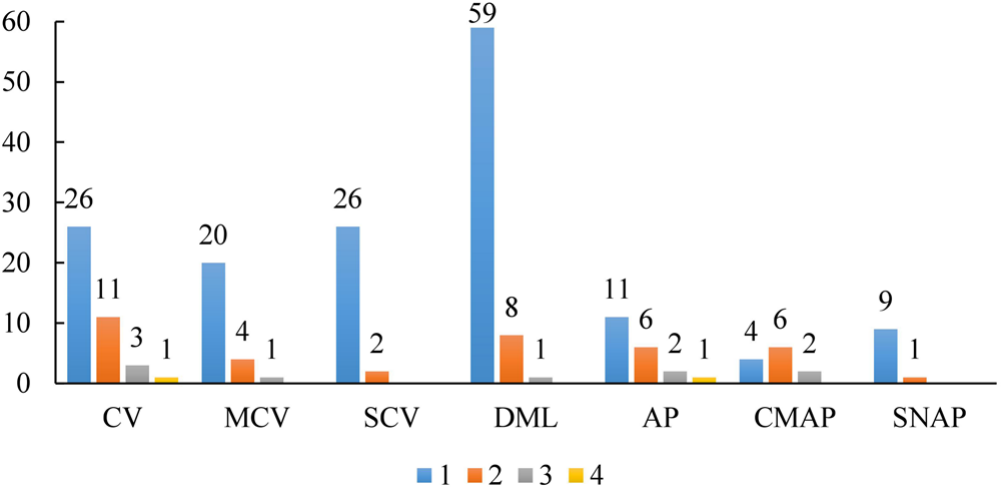
Number of the abnormal parameters of farmers’ peripheral nerve conduction by group. CV, MCV, SCV, DML, AP, CMAP, and SNAP denote the nerve conduction velocity, motor conduction velocity, sensory conduction velocity, distal motor latency, amplitude of the action potential, amplitude of the compound muscle action potential and amplitude of the sensory nerve action potential, respectively.

Figure 2 presents the abnormalities of peripheral nerve conduction. As shown, 41 out of the total 218 sampled farmers had one or more parameters of the nerve conduction velocity falling out of the abnormal range. Among those 41 farmers, 25 and 28 out of them had one or more abnormal parameters of the motor and sensory conduction velocity, respectively. In addition, we found that 68 farmers had one or more abnormal parameters of the distal motor latency. By contrast, only 20 farmers had one or more abnormal parameters of the amplitude. In detail, 12 and 10 out of them had abnormal parameter(s) of the amplitude of compound muscle action potential and sensory nerve action potential, respectively.

### Effect of pesticide use on the abnormalities of peripheral nerve conduction

Tables 2 and 3 provide the adjusted ORs and IRRs with 95% CIs for the occurrence risks of the abnormalities of peripheral nerve conduction. After adjusting for the confounding factors, neither glyphosate nor non-glyphosate herbicides was significantly associated with increase in the odds and incidence rates of the abnormalities of peripheral nerve conduction.

**Table 2:** Adjusted odds ratios (ORs) for the abnormalities of peripheral nerve conduction associated with agricultural pesticide use.

	Nerve conduction velocity			Distal motor latency	Amplitude
	Overall	Motor	Sensory		
Herbicides					
Glyphosate	0.70	1.34	0.64	1.05	1.21
	(0.38, 1.30)	(0.30, 6.03)	(0.35, 1.18)	(0.81, 1.37)	(0.75, 1.97)
Non-glyphosate	0.84	1.46	0.84	1.08	1.21
	(0.62, 1.12)	(0.69, 3.09)	(0.62, 1.14)	(0.90, 1.30)	(0.81, 1.82)
Insecticides & fungicides					
Organophosphorus	1.51**	1.76**	1.43**	1.00	0.95
	(1.21, 1.88)	(1.24, 2.50)	(1.14, 1.79)	(0.89, 1.11)	(0.70, 1.28)
Organonitrogen	2.03**	1.20	2.21**	1.15	1.48*
	(1.29, 3.19)	(0.49, 2.92)	(1.41, 3.48)	(0.92, 1.42)	(1.03, 2.12)
Organosulfur	0.49	1.69	0.48	0.92	0.96
	(0.16, 1.51)	(0.64, 4.47)	(0.17, 1.38)	(0.66, 1.28)	(0.25, 3.61)
Pyrethroid	0.14	0.02	3.57	1.47	2.66
	(0.00, 29.43)	(0.00, 448.25)	(0.06, 205.04)	(0.28, 7.77)	(0.02, 309.15)
Other	1.74	1.68	1.53	0.80	0.80
	(1.00, 3.03)	(0.72, 3.90)	(0.99, 2.35)	(0.54, 1.19)	(0.28, 2.30)
Pseudo *R*^2^	0.67	0.83	0.57	0.11	0.64
Log Likelihood	−105.38	−77.65	−83.58	−135.30	−66.83
Number of observations	218	218	218	218	218

Logistic regression analyses were used to estimate the adjusted ORs. Figures in the parentheses are 95% confidence interval (CI). **p < 0.01, and *p < 0.05. Please see Table S2 for details.

**Table 3:** Adjusted incidence rate ratios (IRRs) for the abnormalities of peripheral nerve conduction associated with agricultural pesticide use.

	Nerve conduction velocity			Distal motor latency	Amplitude		
	Overall	Motor	Sensory		Overall	Motor	Sensory
Herbicides							
Glyphosate	0.86	1.11	0.74	1.02	0.96	1.25	1.04
	(0.67, 1.10)	(0.81, 1.53)	(0.52, 1.06)	(0.85, 1.22)	(0.65, 1.43)	(0.67, 2.34)	(0.49, 2.19)
Non-glyphosate	0.96	0.91	1.01	1.04	1.13	0.32	1.26
	(0.85, 1.08)	(0.76, 1.11)	(0.86, 1.18)	(0.92, 1.17)	(0.86, 1.49)	(0.02, 4.51)	(0.94, 1.69)
Insecticides & fungicides							
Organophosphorus	1.15**	1.22**	1.11	0.99	1.05	1.08	0.92
	(1.07, 1.23)	(1.09, 1.38)	(1.00, 1.23)	(0.92, 1.08)	(0.91, 1.20)	(0.85, 1.38)	(0.65, 1.30)
Organonitrogen	1.17*	0.83	1.26*	1.03	1.36*	1.00	1.38
	(1.01, 1.36)	(0.58, 1.18)	(1.05, 1.52)	(0.92, 1.16)	(1.05, 1.75)	(0.53, 1.86)	(0.93, 2.06)
Organosulfur	0.99	1.28	1.04	0.98	0.67	0.95	1.12
	(0.71, 1.37)	(0.92, 1.80)	(0.67, 1.60)	(0.78, 1.23)	(0.35, 1.25)	(0.34, 2.66)	(0.26, 4.75)
Pyrethroid	0.91	0.16	1.93	1.28	2.75	0.68	3.32
	(0.13, 6.30)	(0.00, 8.38)	(0.16, 22.75)	(0.37, 4.37)	(0.09, 82.63)	(0.00, 450.89)	(0.01, 811.65)
Other	1.16	1.12	1.22	0.97	0.96	0.85	0.94
	(0.95, 1.42)	(0.85, 1.50)	(0.89, 1.69)	(0.76, 1.24)	(0.45, 2.06)	(0.14, 5.17)	(0.22, 3.98)
Pseudo *R*^2^	0.40	0.57	0.35	−165.50	0.51	0.63	0.58
Log Likelihood	−144.37	−92.93	−90.89	218	−89.52	−62.74	−43.95
Number of observations	218	218	218		218	218	218

Negative binomial regression analyses were used to estimate the adjusted IRRs. Figures in the parentheses are 95% confidence interval (CI). **p < 0.01, and *p < 0.05. Please see Table S3 for details.

In terms of insecticides and fungicides, agricultural use of organophosphorus compounds was found to be significantly associated with increased risk of abnormality of nerve conduction velocity. After the confounding factors were adjusted, each kg increase in agricultural use of organophosphorus compounds was likely to induce a 51% (ORs = 1.51; 95% CI: 1.21, 1.88) and 15% (IRRs = 1.15; 95% CI: 1.07, 1.23) increase in the odds and incidence rates of the abnormalities of the nerve conduction velocity, respectively (Tables 2 and 3). In contrast, agricultural use of organophosphorus insecticides and fungicides increased greater risk of the abnormalities of the motor conduction velocity than the sensory conduction velocity. The odd ratios for the abnormalities of the motor and sensory conduction velocity associated with agricultural use of organophosphorus insecticides and fungicides were 1.76 (95% CI: 1.24, 2.50) and 1.43 (95% CI: 1.14, 1.79), respectively (Tables 2 and 3). Moreover, a 22% increase in the incidence rates (IRRs = 1.22; 95% CI: 1.09, 1.38) for the abnormalities of the motor conduction velocity was observed for each kg increase in agricultural use of organophosphorus insecticides and fungicides (Tables 2 and 3). However, there was not significant association of organophosphorus compounds with increased incidence rates for the abnormalities of the sensory conduction velocity.

There was also significant association between increased risk of the abnormalities of peripheral nerve conduction and agricultural use of organonitrogen insecticides and fungicides. As shown in Tables 2 and 3, each kg increase in agricultural use of organonitrogen insecticides and fungicides was significantly associated with a 103% (ORs = 2.03; 95% CI: 1.29, 3.19) increase in the odds of the abnormalities of the nerve conduction velocity. Moreover, the incidence rates of the abnormalities of the nerve conduction velocity also increased by 17% (IRRs = 1.17; 95% CI: 1.01, 1.36) for each kg increase in agricultural use of organonitrogen compounds. Furthermore, each kg increase in agricultural use of organonitrogen compounds significantly induced a 121% (ORs = 2.21; 95% CI: 1.41, 3.48) and 26% (IRRs = 1.26; 95% CI: 1.05, 1.52) increase in the odds and incidence rates of the abnormalities of the sensory conduction velocity, respectively (Tables 2 and 3). In addition, agricultural use of organonitrogen insecticides and fungicides also increased the odds (ORs = 1.48; 95% CI: 1.03, 2.12) and incidence rates (IRRs = 1.36; 95% CI: 1.05, 1.75) of the abnormalities of the compound muscle and sensory nerve action potential amplitudes.

In terms of the other classes of insecticides and fungicides, we found that agricultural uses of organosulfur and pyrethroid compounds were not significantly associated with the abnormalities of peripheral nerve conduction.

## Discussion

Using logistic and negative binomial regression analyses, this study demonstrated that one-year agricultural uses of different classes of pesticides induced different effects on the abnormalities of farmers’ peripheral nerve conduction in China under the actual field conditions. We found that neither glyphosate nor non-glyphosate herbicides used by the sampled farmers was significantly associated with increased risk of the abnormalities of peripheral nerve conduction. In sharp contrast, agricultural uses of organophosphorus and organonitrogen insecticides and fungicides would significantly induce the abnormalities of farmers’ peripheral nerve conduction.

In terms of herbicides, we found that neither glyphosate nor non-glyphosate herbicides used by the sampled farmers was significantly associated with increased risk of the abnormalities of farmers’ peripheral nerve conduction. The results was consistent with our previous study in which neither glyphosate nor non-glyphosate herbicides was found to induce the marginal changes in parameters of peripheral nerve conduction^8^. In the existing literature, glyphosate has often been considered as one of the least toxic pesticides widely used worldwide^10,11^. However, a report released by the International Agency for Research on Cancer (IARC) in 2015 classified glyphosate as probably human carcinogens, which gave birth to an immediate debate^12–14^. Moreover, there is little evidence on the adverse effects of glyphosate use under the actual field conditions on farmers’ peripheral nerve conduction. However, the results in this study were insufficient to tell a perfect story about the effects of the use of glyphosate on peripheral nerve conduction. The key reason is that the amount of glyphosate used by the sampled farmers might be relatively too low to induce the observable abnormalities.

In terms of insecticides and fungicides, agricultural uses of organophosphorus and organonitrogen compounds would significantly increase the risk of abnormalities of peripheral nerve conduction, while organosulfur and pyrethroid compounds used by the sampled farmers were not associated with increased risk of the abnormalities of peripheral nerve conduction. The significant association between the abnormalities of nerve conduction velocity and agricultural use of organophosphorus insecticides and fungicides demonstrated that organophosphorus insecticides and fungicides would induce the nerve conduction velocity to fall below the normal range, which is an observable signal of increased risk of demyelination disease. The results in this study were consistent with most previous studies^3,15–17^. By contrast, agricultural use of organonitrogen insecticides and fungicides was significantly associated with not only increased risk of demyelination disease but axonal damages of farmers’ peripheral nerves. The increased risk of demyelination disease induced by agricultural use of organonitrogen insecticides and fungicides was mainly associated with the sensory nerve conduction. Moreover, we also found that agricultural use of organonitrogen insecticides and fungicides would induce the amplitude to fall below the normal range, which was a signal of axonal damage to peripheral nerves. In the existing literature, many widely used organonitrogen insecticides and fungicides, such as carbamate and neonicotinoid compounds, were considered to induce severe neurotoxicity^18^. In contrast, agricultural uses of organosulfur and pyrethroid insecticides and fungicides were not associated with the abnormalities of farmers’ peripheral nerve conduction.

Our results in this study have important implications for reducing pesticide use in China. To mitigate the adverse effects of substantial pesticide use, some highly toxic pesticides have been prohibited in crop production in China^19^. However, many organophosphorus and organonitrogen insecticides and fungicides with relative high toxicity, such as turbufos, omethoate, phorate, isocarbophos, acetamiprid and imidacloprid, remain frequently and widely used. In the context, this study could emphasize the importance of substituting the low-toxic and health-friendly pesticides for those relative-high-toxic organophosphorus and organonitrogen insecticides and fungicides.

The strengths and weaknesses coexist in this study. The literature on the effects of agricultural pesticide use on farmers’ peripheral nerve conduction under the actual field conditions is still rare. This study sorted out the association of agricultural uses of different classes of pesticides with the abnormalities of peripheral nerve conduction. In the context, the results of this study may contribute to the existing literature by providing the evidence of neurological effects of pesticide exposure under the conditions of agricultural production. However, the duration for assessing pesticide use was confined to one year, which may limit the interpretation since such neurological effect may be chronic. In the context, the association of agricultural pesticide use with the abnormalities of peripheral nerve conduction should be investigated in a prolonged duration. In addition, the association between F waves in peripheral nerve conduction and agricultural pesticide use deserves further investigation.

In conclusion, agricultural uses of different classes of pesticides may induce different effects on farmers’ peripheral nerve conduction. Specifically, the results showed that neither glyphosate and non-glyphosate herbicides used by the sampled farmers would not increase risk of the abnormalities of peripheral nerve conduction under the actual field conditions. In sharp contrast, agricultural use of organophosphorus insecticides and fungicides was associated with increased risk of the abnormalities of the nerve conduction velocity. In other words, organophosphorus compounds would increase the risk of demyelination disease and further reduce the nerve conduction velocity. Similarly, agricultural use of organonitrogen insecticides and fungicides was also associated with increased risk of demyelination disease and reduction of the nerve conduction velocity (mainly the sensory nerve conduction). Moreover, we also found that organonitrogen insecticides and fungicides used by the sampled farmers would also induce the abnormalities of amplitude of compound muscle and sensory nerve action potential, which is a signal of axonal damages of peripheral nerves. However, there was not significant association of organosulfur or pyrethroid insecticides and fungicides with the abnormalities of peripheral nerve conduction.

## Electronic supplementary material

Supplementary Tables
