# Supplementary material for: A comparison of the effects of agricultural pesticide uses on peripheral nerve conduction in China
Source: Sci Rep. 2018 Jun 25;8:9621. doi: 10.1038/s41598-018-27713-6 (PMC6018562; doi:10.1038/s41598-018-27713-6)
Supplement: Supplementary file 1 — Supplementary Tables [file 41598_2018_27713_MOESM1_ESM.docx]

**Supplemental Tables**

**A comparison of the effects of agricultural pesticide uses on peripheral nerve conduction in China**

**Authors:**

**Chao Zhang, Yiduo Sun, Ruifa Hu, Jikun Huang, Xusheng Huang, Yifan Li, Yanhong Yin, and Zhaohui Chen**

**Table of Contents**

**Table S1.** Normal range of parameters of peripheral nerve conduction

**Table S2.** Adjusted odds ratios (ORs) for the abnormalities of peripheral nerve conduction

**Table S3.** Adjusted incidence rate ratios (IRRs) for the abnormalities of peripheral nerve conduction

**Table S1.** Normal range of parameters of peripheral nerve conduction

| Definition | Unit | Normal range |
| --- | --- | --- |
| **Nerve conduction velocity** |  |  |
| **Motor conduction velocity** |  |  |
| Median Nerve | m/s | ≥50 |
| Ulnar Nerve | m/s | ≥50 |
| Tibial Nerve | m/s | ≥40 |
| Common Peroneal Nerve | m/s | ≥45 |
| **Sensory conduction velocity** |  |  |
| Median Nerve | m/s | ≥50 |
| Ulnar Nerve | m/s | ≥50 |
| Sural Nerve | m/s | ≥50 |
|  |  |  |
| **Distal motor latency** |  |  |
| Median Nerve | ms | ≤3.63 |
| Ulnar Nerve | ms | ≤3.07 |
| Tibial Nerve | ms | ≤4.80 |
| Common Peroneal Nerve | ms | ≤4.50 |
|  |  |  |
| **Amplitude** |  |  |
| **Compound muscle action potential** |  |  |
| Median Nerve (Proximal) | mV | ≥5.0 |
| Median Nerve (Distal) | mV | ≥5.0 |
| Ulnar Nerve (Proximal) | mV | ≥5.0 |
| Ulnar Nerve (Distal) | mV | ≥5.0 |
| Tibial Nerve (Proximal) | mV | ≥4.8 |
| Tibial Nerve (Distal) | mV | ≥4.8 |
| Common Peroneal Nerve (Proximal) | mV | ≥2.0 |
| Common Peroneal Nerve (Distal) | mV | ≥2.0 |
| **Sensory nerve action potential** |  |  |
| Median Nerve | mV | ≥2.0 |
| Ulnar Nerve | mV | ≥2.0 |
| Sural Nerve | mV | ≥2.0 |

**Table S2.** Adjusted odds ratios (ORs) for the abnormalities of peripheral nerve conduction

|  | Nerve conduction velocity | | | Distal motor latency | Amplitude |
| --- | --- | --- | --- | --- | --- |
|  | Overall | Motor | Sensory |  |  |
| **Herbicides** |  |  |  |  |  |
| Glyphosate | 0.70 | 1.34 | 0.64 | 1.05 | 1.21 |
|  | (0.38,1.30) | (0.30,6.03) | (0.35,1.18) | (0.81,1.37) | (0.75,1.97) |
| Non-glyphosate herbicides | 0.84 | 1.46 | 0.84 | 1.08 | 1.21 |
|  | (0.62,1.12) | (0.69,3.09) | (0.62,1.14) | (0.90,1.30) | (0.81,1.82) |
| **Insecticides & fungicides** |  |  |  |  |  |
| Organophosphorus | 1.51^**^ | 1.76^**^ | 1.43^**^ | 1.00 | 0.95 |
|  | (1.21,1.88) | (1.24,2.50) | (1.14,1.79) | (0.89,1.11) | (0.70,1.28) |
| Organonitrogen | 2.03^**^ | 1.20 | 2.21^**^ | 1.15 | 1.48^*^ |
|  | (1.29,3.19) | (0.49,2.92) | (1.41,3.48) | (0.92,1.42) | (1.03,2.12) |
| Organosulfur | 0.49 | 1.69 | 0.48 | 0.92 | 0.96 |
|  | (0.16,1.51) | (0.64,4.47) | (0.17,1.38) | (0.66,1.28) | (0.25,3.61) |
| Pyrethroid | 0.14 | 0.02 | 3.57 | 1.47 | 2.66 |
|  | (0.00,29.43) | (0.00,448.25) | (0.06,205.04) | (0.28,7.77) | (0.02,309.15) |
| Other | 1.74 | 1.68 | 1.53 | 0.80 | 0.80 |
|  | (1.00,3.03) | (0.72,3.90) | (0.99,2.35) | (0.54,1.19) | (0.28,2.30) |
| **Characteristics** |  |  |  |  |  |
| Age | 1.13^*^ | 1.33^*^ | 1.14^**^ | 1.06^**^ | 1.21^*^ |
|  | (1.02,1.25) | (1.01,1.74) | (1.04,1.26) | (1.02,1.10) | (1.03,1.41) |
| Male | 3.27 | 8.10 | 3.27 | 0.89 | 1.48 |
|  | (0.39,27.34) | (0.15,435.43) | (0.34,31.06) | (0.37,2.13) | (0.12,17.85) |
| BMI | 1.14 | 0.72 | 1.17 | 1.05 | 1.42^*^ |
|  | (0.89,1.45) | (0.45,1.16) | (0.91,1.50) | (0.95,1.17) | (1.01,2.01) |
| Smoking habit | 2.51 | 1.06 | 4.07 | 1.07 | 1.64 |
|  | (0.50,12.49) | (0.05,22.39) | (0.65,25.40) | (0.50,2.29) | (0.19,14.44) |
| Alcohol consumption | 0.67 | 0.41 | 0.60 | 1.33 | 0.45 |
|  | (0.16,2.77) | (0.03,5.92) | (0.13,2.77) | (0.66,2.66) | (0.07,3.10) |
| Adoption of protective measures | 2.55 | 48.93 | 1.16 | 0.87 | 1.73 |
|  | (0.31,20.90) | (0.41,5812.65) | (0.14,9.73) | (0.31,2.43) | (0.09,32.62) |
| Diabetes mellitus | 0.18 | 0.49 | 0.49 | 0.87 | 3.32 |
|  | (0.00,7.53) | (0.00,5082.52) | (0.03,8.65) | (0.23,3.24) | (0.16,67.86) |
| **Baseline** | 265.29^**^ | 1530.07^**^ | 220.54^**^ | 1.74 | 166.03^**^ |
|  | (37.21,1891.23) | (45.69,51244.57) | (25.94,1875.00) | (0.89,3.39) | (13.47,2046.01) |
| **Regions** |  |  |  |  |  |
| Hebei | 2.83 | 1.62 | 1.65 | 0.60 | 0.17 |
|  | (0.39,20.54) | (0.06,40.26) | (0.26,10.62) | (0.23,1.53) | (0.01,2.15) |
| Jiangxi | 1.01 | 0.40 | 0.47 | 0.31^*^ | 0.18 |
|  | (0.15,6.61) | (0.01,13.96) | (0.07,3.01) | (0.12,0.84) | (0.02,2.16) |
| Number of observation | 218 | 218 | 218 | 218 | 218 |
| Pseudo *R*^2^ | 0.67 | 0.83 | 0.57 | 0.11 | 0.64 |
| Log Likelihood | -105.38 | -77.65 | -83.58 | -135.30 | -66.83 |

Logistic regression analyses were used to estimate the adjusted ORs. Figures in the parentheses are 95% confidence interval (CI). ** *p* < 0.01, and * *p* < 0.05.

**Table S3.** Adjusted incidence rate ratios (IRRs) for the abnormalities of peripheral nerve conduction

|  | Nerve conduction velocity | | | Distal motor latency | Amplitude | | |
| --- | --- | --- | --- | --- | --- | --- | --- |
|  | Overall | Motor | Sensory |  | Overall | Motor | Sensory |
| **Herbicides** |  |  |  |  |  |  |  |
| Glyphosate | 0.86 | 1.11 | 0.74 | 1.02 | 0.96 | 1.25 | 1.04 |
|  | (0.67,1.10) | (0.81,1.53) | (0.52,1.06) | (0.85,1.22) | (0.65,1.43) | (0.67,2.34) | (0.49,2.19) |
| Non-glyphosate herbicides | 0.96 | 0.91 | 1.01 | 1.04 | 1.13 | 0.32 | 1.26 |
|  | (0.85,1.08) | (0.76,1.11) | (0.86,1.18) | (0.92,1.17) | (0.86,1.49) | (0.02,4.51) | (0.94,1.69) |
| **Insecticides & fungicides** |  |  |  |  |  |  |  |
| Organophosphorus | 1.15** | 1.22** | 1.11 | 0.99 | 1.05 | 1.08 | 0.92 |
|  | (1.07,1.23) | (1.09,1.38) | (1.00,1.23) | (0.92,1.08) | (0.91,1.20) | (0.85,1.38) | (0.65,1.30) |
| Organonitrogen | 1.17* | 0.83 | 1.26* | 1.03 | 1.36* | 1.00 | 1.38 |
|  | (1.01,1.36) | (0.58,1.18) | (1.05,1.52) | (0.92,1.16) | (1.05,1.75) | (0.53,1.86) | (0.93,2.06) |
| Organosulfur | 0.99 | 1.28 | 1.04 | 0.98 | 0.67 | 0.95 | 1.12 |
|  | (0.71,1.37) | (0.92,1.80) | (0.67,1.60) | (0.78,1.23) | (0.35,1.25) | (0.34,2.66) | (0.26,4.75) |
| Pyrethroid | 0.91 | 0.16 | 1.93 | 1.28 | 2.75 | 0.68 | 3.32 |
|  | (0.13,6.30) | (0.00,8.38) | (0.16,22.75) | (0.37,4.37) | (0.09,82.63) | (0.00,450.89) | (0.01,811.65) |
| Other | 1.16 | 1.12 | 1.22 | 0.97 | 0.96 | 0.85 | 0.94 |
|  | (0.95,1.42) | (0.85,1.50) | (0.89,1.69) | (0.76,1.24) | (0.45,2.06) | (0.14,5.17) | (0.22,3.98) |
| **Characteristics** |  |  |  |  |  |  |  |
| Age | 1.06** | 1.07* | 1.05 | 1.03* | 1.07* | 1.03 | 1.25* |
|  | (1.02,1.10) | (1.00,1.14) | (1.00,1.11) | (1.01,1.06) | (1.01,1.14) | (0.92,1.14) | (1.02,1.54) |
| Male | 2.93 | 7.17 | 1.23 | 0.80 | 1.97 | 0.40 | 4.92 |
|  | (0.91,9.47) | (0.61,84.64) | (0.35,4.38) | (0.43,1.50) | (0.37,10.42) | (0.00,38.57) | (0.26,92.79) |
| BMI | 1.02 | 0.84 | 1.06 | 1.02 | 1.19* | 1.29 | 0.83 |
|  | (0.91,1.14) | (0.68,1.03) | (0.92,1.23) | (0.94,1.10) | (1.01,1.39) | (0.95,1.75) | (0.49,1.38) |
| Smoking habit | 1.89 | 0.91 | 2.07 | 0.98 | 1.47 | 18.97 | 0.23 |
|  | (0.85,4.19) | (0.26,3.18) | (0.72,5.92) | (0.54,1.75) | (0.32,6.76) | (0.16,2190.29) | (0.01,3.81) |
| Alcohol consumption | 0.74 | 0.58 | 1.19 | 1.31 | 0.35 | 0.04 | 0.95 |
|  | (0.39,1.41) | (0.22,1.54) | (0.50,2.86) | (0.79,2.18) | (0.09,1.44) | (0.00,1.10) | (0.13,6.81) |
| Adoption of protective measures | 1.59 | 1.57 | 1.07 | 1.06 | 0.78 | 0.63 | 0.00 |
|  | (0.67,3.78) | (0.50,4.95) | (0.23,5.06) | (0.51,2.20) | (0.14,4.28) | (0.06,6.84) | (0.00,.) |
| Diabetes mellitus | 0.50 | 0.55 | 0.80 | 0.91 | 1.33 | 5.14 | 0.00 |
|  | (0.08,3.02) | (0.01,20.21) | (0.09,7.27) | (0.31,2.65) | (0.13,13.23) | (0.17,155.77) | (0.00,.) |
| **Baseline** | 3.59** | 17.17** | 10.67** | 1.47** | 6.02** | 21.02** | 6.26 |
|  | (2.40,5.39) | (5.67,52.02) | (3.94,28.87) | (1.13,1.92) | (3.12,11.62) | (3.82,115.75) | (0.70,56.24) |
| **Regions** |  |  |  |  |  |  |  |
| Hebei | 1.58 | 3.11 | 1.66 | 0.84 | 0.67 | 0.19 | 0.46 |
|  | (0.69,3.63) | (0.78,12.37) | (0.47,5.84) | (0.44,1.60) | (0.16,2.79) | (0.01,4.16) | (0.03,8.19) |
| Jiangxi | 0.90 | 1.49 | 1.26 | 0.61 | 0.81 | 0.24 | 1.32 |
|  | (0.38,2.14) | (0.36,6.22) | (0.40,3.98) | (0.31,1.20) | (0.24,2.77) | (0.03,2.29) | (0.13,14.00) |
| Number of observation | 218 | 218 | 218 | 218 | 218 | 218 | 218 |
| Pseudo *R*^2^ | 0.40 | 0.57 | 0.35 | 0.09 | 0.51 | 0.63 | 0.58 |
| Log Likelihood | -144.37 | -92.93 | -90.89 | -165.50 | -89.52 | -62.74 | -43.95 |

Negative binomial regression analyses were used to estimate the adjusted IRRs. Figures in the parentheses are 95% confidence interval (CI). ** *p* < 0.01, and * *p* < 0.05.
